# Traditional ecological knowledge and non-food uses of stingless bee honey in Kenya’s last pocket of tropical rainforest

**DOI:** 10.1186/s13002-023-00614-3

**Published:** 2023-09-28

**Authors:** Madeleine Héger, Pierre Noiset, Kiatoko Nkoba, Nicolas J. Vereecken

**Affiliations:** 1 Agroecology Lab, Université Libre de Bruxelles (ULB), Boulevard du Triomphe CP 264/02, 1050 Brussels, Belgium; 2 International Centre of Insect Physiology and Ecology (Icipe), P.O. Box 30772-00100, Nairobi, Kenya

**Keywords:** Indigenous knowledge, Meliponini, Kakamega forest, Traditional medicine

## Abstract

**Background:**

Stingless bee honey (SBH) is a natural remedy and therapeutic agent traditionally used by local communities across the (sub-)tropics. Forest SBH represents a prime non-timber forest product (NTFP) with a potential to revitalize indigenous foodways and to generate income in rural areas, yet it is also used in a variety of non-food contexts that are poorly documented in sub-Saharan Africa and that collectively represent a significant part of the local traditional ecological knowledge (TEK) passed on across generations. Documenting TEK of local communities in African tropical forests facing global change is a pressing issue to recognize the value of their insights, to evaluate their sustainability, to determine how they contribute to enhancing conservation efforts, and how TEK generally contributes to the well-being of both the natural environment and the communities that rely on it. This is particularly important to achieve in Kenya’s only tropical rainforest at Kakamega where SBH production and non-food uses have evolved and diversified to a remarkable extent.

**Methods:**

We used ethnographic techniques and methods, including semi-structured questionnaires and recorded interviews. We used snowball sampling, a non-probability sampling method where new interviewees were recruited by other respondents, to collectively form a sample consisting of 36 interviewees (including only one woman).

**Results:**

Our results indicate that local communities in Kakamega were able to discriminate between six different and scientifically recognized stingless bee species, and they provided detailed accounts on the species-specific non-food uses of these SBH. Collectively, we recorded an array of 26 different non-food uses that are all passed on orally across generations in the Kakamega community.

**Conclusion:**

Our results uncover the vast and hitherto unexpected diversity of TEK associated with SBH and pave the way for a systematic survey of SBH and their non-food uses across a network of communities in different environments and with different cultural backgrounds in the Afrotropics. This, along with parallel and more in-depth investigations into honey chemistry, will help develop a comprehensive understanding of SBH, offering insights into holistic ecosystem management, resilience and adaptation while in the mid- to long-term promoting cross-cultural exchanges and pathways for the revitalization of cultural practices and traditions.

**Supplementary Information:**

The online version contains supplementary material available at 10.1186/s13002-023-00614-3.

## Science highlights

- Uncovering the hitherto unexpected diversity of stingless bee honeys and their non-food uses in Kakamega Forest (Kenya).
- A total of 26 different non-food uses were recorded (primarily medicinal, traditional, and spiritual), some of which are species-specific to certain stingless bee species in this tropical forest fragment.
- Knowledge on the uses of SBH is ancestral and is shared orally across generations, with potential for cross-cultural exchanges as well as the revitalization of cultural practices and traditions.

**Supplementary Information:**

The online version contains supplementary material available at 10.1186/s13002-023-00614-3.

## Background

The relationship between local communities and the forest in the Afrotropics is deeply intertwined, shaped by centuries of coexistence, dependence, and cultural significance. This relationship is multifaceted and encompasses ecological, social, economic, and spiritual dimensions that are key to rural livelihoods [1]. Tropical forests represent an increasingly exploited source of wooden material, but also of so-called non-timber forest products (NTFP) or resources derived from forests that are not primarily harvested for their wood or timber, such as animals, edible and medicinal plants and by-products [2]. Over time, local communities have gained intimate empirical and ecological knowledge of the forest and its ecosystems [3]. This “traditional ecological knowledge” (TEK) is usually passed down orally through generations and plays a vital role in guiding community decisions and practices, bearing witness to the biological and cultural diversity of our planet [4]. As the world is facing a rapid erosion of this valuable natural and cultural heritage, due to decades of colonialism, agricultural intensification and shifting of land uses causing deforestation among other forms of anthropogenic environmental disturbance in a context of food system globalization, we are likely to witness an increased decline in communities living close to or within tropical forests [4]. This, in turn, will contribute to obliterating their cultural identities, and lead to the disappearance of TEK, including aspects of subsistence, habitat protection, spiritual significance, and traditional or folk medicine among others [3, 5].

Worldwide, natural remedies derived from insects and their by-products have long been used in traditional or folk healthcare [6]. Honey produced by the iconic Western Honey Bee, *Apis mellifera*, is one of these natural substances and has been gathered in Nature since ancient times [7] and praised for its medicinal and nutritional properties [8]. Interestingly, there is another group of less known honey producing social bees, called stingless bees (Hymenoptera: Apidae: Meliponini) that are particularly associated with indigenous forest habitats and found across the (sub-)tropical regions of the world [9]. Stingless bee honey (SBH) has gained academic recognition and even integration into mainstream healthcare systems in some countries, owing to its anti-inflammatory, anti-viral/fungal, anticancer, and antioxidant properties [10] among others that also make SBH useful in a range of applications beyond its culinary uses [11].

Non-food uses of SBH are particularly developed in East Africa, including in Kenya, where SBH is informally reported to be commonly used as medicine, food and in traditional rituals [12]. In Kenya, domesticated species such as *Meliponula bocandei* produces up to 5 kg of honey; while *M. togoensis* produces 3 kg of honey and *M. ferruginea* produces 2 kg of honey. The cost of 1 kg of SBH in Kenya varies between USD 15 to USD 30 depending on either purchased from the local communities or urban areas, respectively. This, in turn, offers the potential to sustain livelihoods through diversification, to secure food and medicine provisions, to revitalize indigenous foodways and to safeguard TEK base in African (sub-)tropical forests [13, 14].

In this study, we aim to contribute to the body of ethnobiological research by documenting and discussing TEK relative to (i) stingless bees (ii) their honeys and (iii) non-food uses of SBH by the local communities living around the Kakamega forest in Western Kenya. Our goal was specifically to investigate and characterize the diversity of non-food uses of SBH, with the perspective to hybridize our surveys with advanced analyses of honey chemistry across a wider network of communities and thereby develop a comprehensive understanding of SBH composition, variation and uses in the Afrotropics.

## Methods

### Study area

**Fig. 1:**
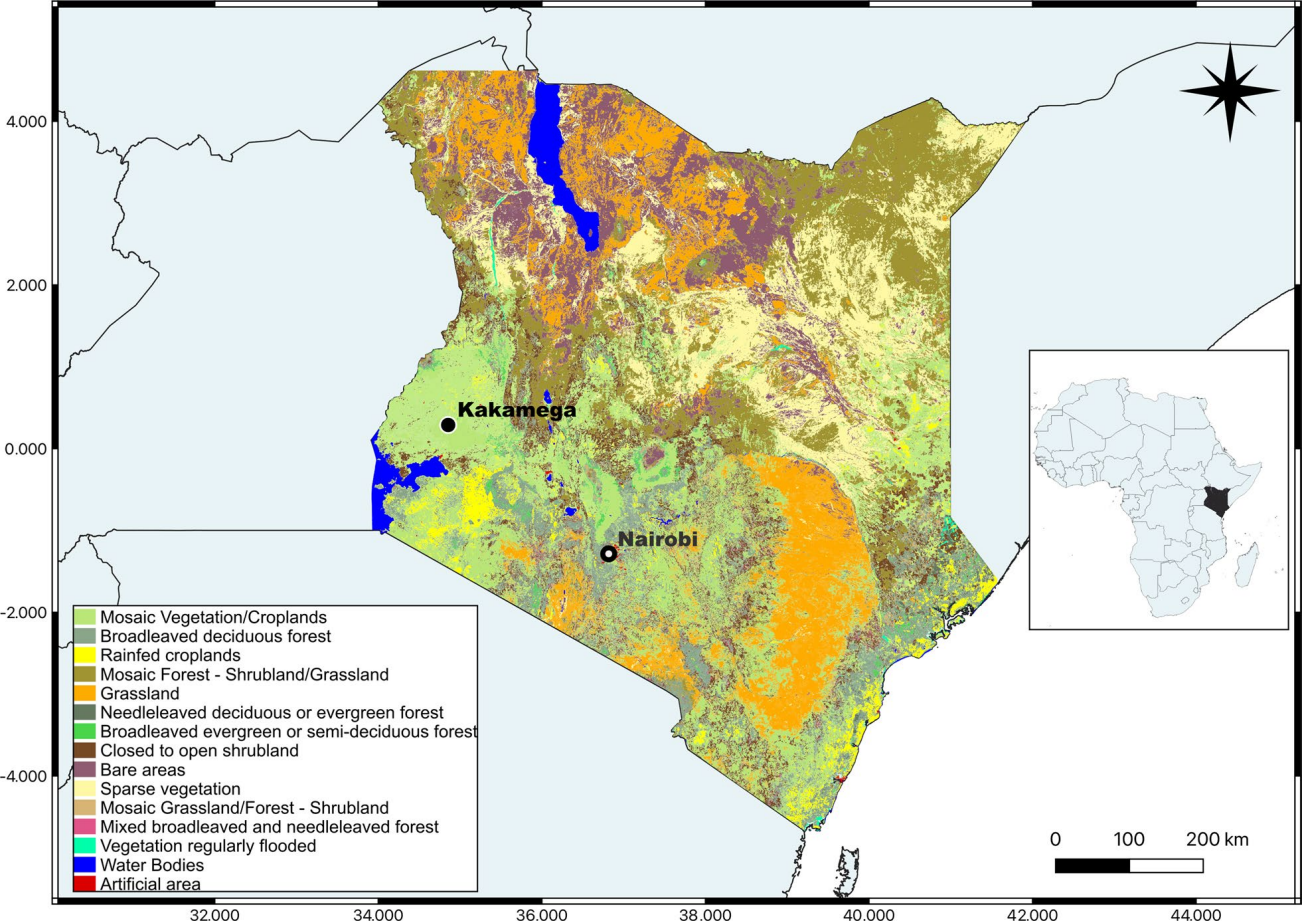
Land cover of Kenya. Map developed using QGIS Version 3.32.2 with geographical data obtained from FAO “Land Cover Land Use of Kenya” dataset

The research took place in Kakamega Forest reserve (0° 09′ N, 34° 50′ E) in Western Kenya (Fig. 1), 40 km northeast of Lake Victoria. It is the only surviving tropical rainforest in the country and last remnant of the ancient Guineo-Congolian rainforest that once spanned the African continent [14]. The forest receives an average rainfall of approximately 2000 mm annually, and the temperature are constant throughout the year, with mean daily minimum and maximum of 11 °C and 26 °C respectively [15]. The Kakamega forest is located at approximately 1600 m above sea level. It is home to a biodiversity of fauna and flora [16] and is a major hot spot for stingless bees (SB) diversity. The main ethnic group in Western Kenya are Luhya and are divided in subgroups.

Even though the forest represents a highly valuable source of food, wooden materials and herbal medicine for local communities, its area has been halved over the past few years because of unsustainable pressure on resources and a lack of environmentally-friendly and sustainable alternatives for local community income generation [15]. To overcome this situation, the International Center of Insect Physiology and Ecology (*icipe*, Nairobi, Kenya), started working with local communities one decade ago to find ways to replace exploitative and destructive harvesting methods with well-designed hives and suitable stingless bee beekeeping management practices [17]. This explains why meliponiculture (i.e., beekeeping with stingless bees) is well developed in the area, which contributes in diversifying and improving the livelihoods of local communities while conserving nature. It also supports pastoralist activities with ecological application such as pollination that enhance crop productivity [14, 17].

### Surveys

The study was conducted by the authors (see Contributions) in March and April 2022 with questionnaire surveys and in-depth interviews, lasting up to one hour. Some interviews were performed in English, others required translation assistance provided by an in-between person fluent in English, Swahili, and Luhya. The survey (Appendix A) was divided into four sections or over-arching themes, namely: (1) personal information of meliponiculturists (i.e., stingless bee beekeepers) for future reference, (2) extent of knowledge on stingless bee species and diversity, (3) TEK and uses of SBH, (4) personal experience with meliponiculture. Details on these four sections and what they entail are provided in Appendix A. In total, we performed 36 interviews with meliponiculturists and SBH hunters through contacts established within the lively pre-existing network established by our research partners at *icipe*, and we used the snowball sampling method [18] to include more interviewees outside of *icipe*’s network. Non-food uses of SBH were transcribed and recorded for each stingless bee species in an Excel spreadhseet (Additional file 1). Prior to the interviews, we obtained consent from the local authorities to conduct the study. The respondents were informed about the aim of the study and the data collection procedures and voluntarily joined the study [19]. They provided their informed consent for the use of recording devices. The Ethical Committee at *icipe* approved the study.

## Results

All respondents to our survey were Kenyan men except one woman, and they were all ranging in age from 18 to 80 years old. Small-scale farming in rural villages is the main activity of respondents, but some of them were also hunters or employees in small local companies.

The respondents demonstrate their ability to discriminate seven different stingless bee species based on their morphological traits, their nesting habits and even characteristics of the honey they produce. All seven species are found locally, and they are referred to by respondents using local names (Table 1). In the context of Kakamega, meliponiculturists described the support received by hunters who have always been involved in the harvesting of honey. This practice is deeply rooted in the local culture, where honey was traditionally consumed when discovered, and the bee colonies were left to perish. These days, hunters sell the colonies to meliponiculturists instead.

**Table 1:** Overview of seven stingless bee species, their local name(s) and literal meaning(s) in the local language (Luhya) of the community at Kakamega forest in Kenya

Scientific name	Local name(s) in Luhya	Literal meaning in Luhya
*Meliponula (Meliponula) bocandei* (Spinola 1853)	Igora	Igora = lost: “getting lost”; “a lost sweetness in the forest”
*Meliponula (Axestotrigona) ferruginea* (Lepeletier 1841)	Inasasa (Isukha community)	Msaza = man: “men energy”; "sweet"; “found in trees”
*Meliponula (Axestotrigona) togoensis* (Stadelmann 1895)	Iwera (Tiriki community) Iwere (Isukha community)	" medicine"; “clears the stomach”; “not easy to find”
*Hypotrigona* spp. Cockerell 1934	Vuyiyi	“aggressive”; “stupid”; “small”; " found in the walls of the house"
*Liotrigona* Moure 1961	Vuyiyi (Tiriki community) Chihiyi (Isukha community)	Those two words refer to the sound they make “iiiiii”
*Meliponula (Meliplebeia) lendliana* (Friese 1900)	Indakala	"found in the ground"
*Plebeina armata* (Magretti 1895)	Vusitsi	Vusitsi = “wild banana” (found on plantain trees)

**Fig. 2:**
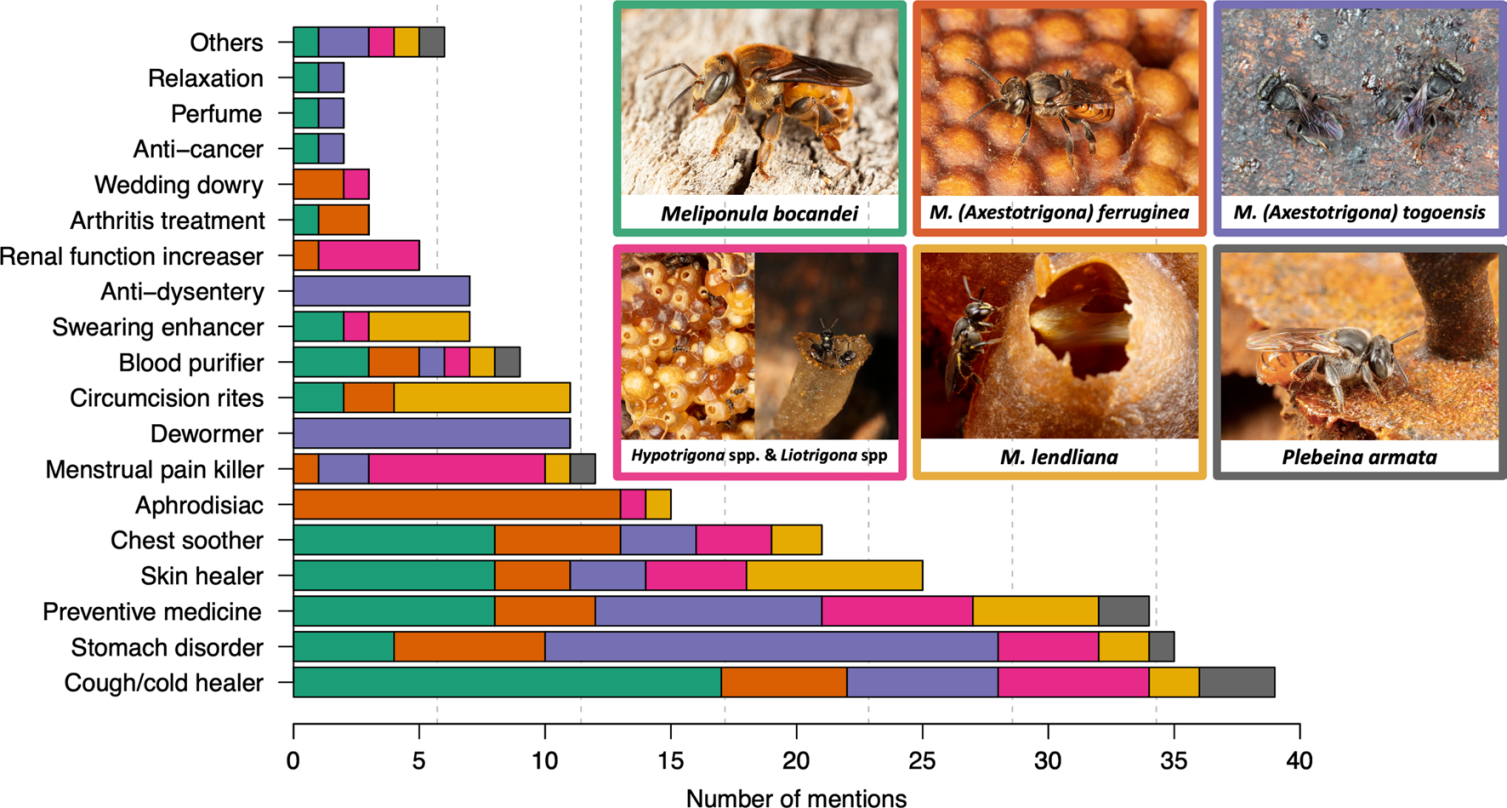
Non-food use attributed regarding the different SB species. Photos © NJ Vereecken

Our results also illustrate that non-food uses have evolved and diversified to a remarkable extent in Kakamega, with a total of 26 claimed uses of SBH. It is worth noting that while most of these non-food uses are related to healthcare, some of them also hold a traditional and spiritual significance. Medicinally, honey is commonly used as a natural remedy to treat various ailments. Traditionally, honey is often incorporated into cultural ceremonies and cultural traditions. Spiritually, honey holds symbolic significance rooted in local beliefs. These categories are not rigid and can overlap. For example, honey from *M. lendliana* plays an essential role in circumcision ceremonies for medicinal purposes as well as during the traditional and spiritual part of the ceremony. In addition, certain species have specific uses. For instance, honey derived from *M. ferruginea* is praised for its aphrodisiac effects, while honey produced by *M. togoensis* is renowned for its effectiveness in alleviating dysentery, treating stomach ailments, and even serving as a dewormer (Fig. 2; Table 2).

**Table 2:** Categories of non-food uses of stingless bee honey in the Kakamega forest of Kenya, and the associated details of these uses

Non-food uses of honey	Details of the uses
Stomach disorders	Stomach pain, ulcers, gastritis
Cough/colds	Sore throat, throat infection, flu
Chest soother	Decongestant, soothe airways
Swearing enhancer	To commit oneself, to improve vow/oath
Skin healer	Wounds, scars, burns, cuts, and any kind of skin ailments
Others	Hair growth promoter, fertility enhancer, urinary problem healer, ingredient to prepare brew, to protect the house from bad spirits, appetizer

**Fig. 3:**
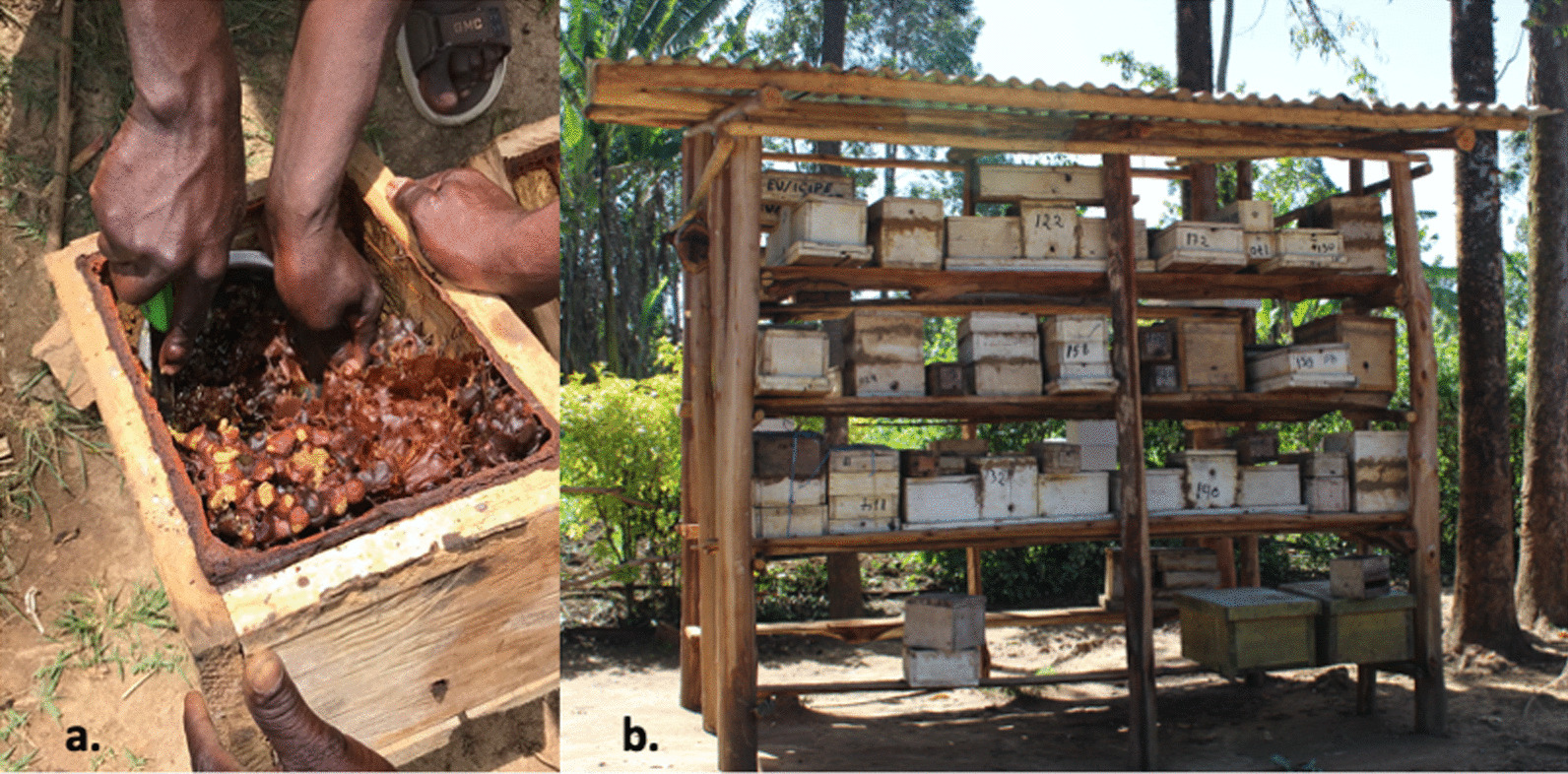
Meliponiculture using man-made structures. a. colony of *Meliponula ferruginea* in a man-made wooden hive b. Meliponarium of one of the respondents, containing stingless bee beehives designed by icipe. Photos © M Héger

The three main reasons why people keep stingless bees in Kakamega are for (1) medicine provision (79%), (2) income generation (74%), and (3) food provision (74%), and the most domesticated species are *M. ferruginea* and *M. togoensis*. According to the participants, they are easy to find in the wild, easy to manage in man-made wooden hives, and they produce high yields of honey (Fig. 3). Despite the well-developed practice of meliponiculture in Kakamega, most respondents (26) still rely on hunting to obtain stingless bee honey. Interestingly, even those who have their own hives predominantly consume honey from wild nests, which highlights the continued prevalence of honey hunting in the region.

## Discussion

In this study, we shed light on hitherto unexplored aspects of the Traditional Ecological Knowledge (TEK) associated with stingless bees and the non-food uses of stingless bee honey (SBH) in Kenya. In line with what has already been documented in other regions of the world [20–22], we report that there is a multitude of uses of the honeys from the different SB species, and that some of these uses are species-specific. Additionally, we observed that the understanding of SB extends beyond the production of honey in the local communities of Kakamega forest. Our results clearly illustrate that SBH serves as a highly regarded therapeutic substance, deeply intertwined with the cultural, traditional, and spiritual aspects of the people’s lives. As stingless bees are perceived as friendly creatures and are commonly encountered in the local environment, people are accustomed to their presence and frequently engage with them.

In Kakamega, meliponiculture is often perceived as a predominantly male occupation, due to the necessity of leaving home and children for extended periods of time, venturing into the forest to find active colonies of stingless bees. The social and cultural norm in Kakamega includes a division of labor between men and women where the latter are responsible for maintaining the household and looking after children, which explains why only one woman was interviewed. In Kaijado County (Kenya), women are prohibited from transporting honey as beekeeping is considered a male-dominated activity [23], and rather gather wood in the forest, which is a common activity for women in this context. Gender roles and division of labor are complex and can vary widely between cultures and communities, and while honey hunting might be perceived as conflicting with women's traditional roles in Kakamega communities, future research should explore how integrating women's perspectives, knowledge, and participation in these activities can lead to more sustainable and equitable outcomes for both communities and the environment.

Several applications of SBH mentioned by the respondents (Table 2) are consistent with previous findings documented by researchers worldwide. Indeed, the treatment of skin ailments, stomach disorders, and respiratory conditions have also been identified in Ecuador [20], Benin [24] and Nepal [25] among other regions. However, not all reports on the non-food uses of SBH are convergent: for example, the pygmy people in Uganda use *M. ferruginea* honey to treat wounds [26] and to alleviate constipation [27], whereas in Kakamega, the same honey is primarily used as an aphrodisiac as mentioned above, and honey from *M. bocandei* and *M. lendliana* are preferred to heal skin problems. Additionally, SBH is commonly utilized for its potential in treating dysentery rather than for alleviating constipation in Kakamega. In Burkina Faso, honey (in general) is also used to alleviate menstrual pain [28], but among the Guayakis in the Chaco region in Argentina, the consumption of honey by women is forbidden during their menstruation, as there is a local belief that it could bring bad luck to the whole community [29]. Similar to Mayan societies [30, 31] honey in Kakamega is employed during rites of passage, particularly during the circumcision ceremony, SBH’s significance lies in its sacred and pure nature, contributing to its spiritual value, which is crucial during oath taking. Honey from *M. lendliana* is preferably used in that context. The well-established antibacterial and anti-inflammatory properties of SBH, as well as its ability to stimulates tissue regeneration, further contribute to its regular use in healthcare.

SBH is found to be a near-perfect Non-Timber Forest Product (NTFP); and in East Africa SB products (SBP) have been sourced since many decades from wild colonies using harvesting practice that destroy nest and therefore occasioning colony loses [13]. Currently, with the increase of knowledge in the domestication of SB in (sub-) tropical region around the world and in East Africa in particular meliponiculturists (SB beekeepers) are capable of responding to higher demand without eroding the resource base, as long as the preferred sources of pollen and nectar are maintained intact [2]. Meliponiculture can contribute as an alternative source of income to improve the livelihoods of rural communities through honey production [30].

## Conclusion

By emphasizing the need for greater focus on analyzing local social and cultural values associated with non-timber forest products (NTFPs), our study sheds light on meliponiculture as an activity that safeguards an important part of the cultural heritage shared within local communities. Meliponiculture is a way to sustain livelihoods, to secure food and medicine provisions, to revitalize indigenous foodways and to safeguard indigenous knowledge base in African tropical forests, while promoting a more sustainable use of natural resources[11]. Beyond the hitherto little explored natural compositional variation of SBH (e.g. [32–34]), many other drivers can explain the depth and persistence of TEK, including the variation in ethnicities, environmental conditions, and degradation, but also the contrasts in regional political history and domination. More fine-grained field surveys and analyses using state-of-the art laboratory techniques should be performed across the Afrotropic to fully document TEK associated with SBH, their variation, their socio-environmental drivers, and the extent of their co-variation with the patterns of bioactive compounds detected in these highly praised honeys.

### Appendix A: supplementary material and methods: questionnaire

1. Personal information
  Name; age; gender
  Address: county, village, GPS coordinates
  Academic level
  Occupation
  Phone number
  Household size + position
  Tribe/community
2. Traditional ecological knowledge on stingless bees
  Do you know stingless bees?
    Which ones?
    Local taxonomy (how you differentiate each species)
    Name in local language meaning
  Which species are mostly found in your area?
  Which species do you domesticate? Why?
  Are stingless bees important to you? Why?
3. Local knowledge and non-food uses of stingless bee honey
  Do you use stingless bee honey? For what? How? Taste characteristics?
  Where do you obtain these hive products from?
  When is the honey used? Special occasion? Daily?
  What role (value) do stingless bee honey play in your culture? Beliefs?
  Do you use other products of the hive? For what? How?
  Where did you get knowledge from? Who did you transfer this knowledge to?
  Have you ever heard of any other possible uses of stingless bee’s honey?
4. Personal experience with stingless beekeeping
  Since how long have you practiced meliponiculture?
  Have you been trained on modern stingless beekeeping? If yes, by who?
  What are your motivating factors to do stingless beekeeping:
  How many stingless bee hives do you have in total?
  In which type of structure do you house the stingless?
  Where do you keep colonies that you domesticate?
  How do you obtain your starter colonies?
    If baiting swarms, what method do you use?
  What are the natural enemies and pests affecting your colonies?
  Are you facing any challenges in keeping stingless bees?
  Quantities of honey harvested per hive per species? After how many months?
  How do you conserve (storage) harvested hive products?
  Do you sell the products? If yes, how much
  Where are the products sold? Locally or out of Kakamega ?
  Who are your clients?
  How do you advertise your products? Online, mouth to mouth, radio, TV…

### Supplementary Information

**Additional file 1.** Non-food uses of honey for each stingless bee species.

## Data Availability

The dataset supporting the conclusions of this article is included within the article (and its additional file(s)).

## Abbreviations

F.R.S-FNRS: Fonds National de la Recherche Scientifique
Icipe: International Centre of Insect Physiology and Ecology
NTFP: Non-timber forest product
PDR: Projet de Recherches
SB: Stingless bee
SBH: Stingless bee honey
SBP: Stingless bee product
TEK: Traditional ecological knowledge
ULB: Université libre de Bruxelles
